# Mechanical Contributions of the Cortical and Trabecular Compartments Contribute to Differences in Age-Related Changes in Vertebral Body Strength in Men and Women Assessed by QCT-Based Finite Element Analysis

**DOI:** 10.1002/jbmr.287

**Published:** 2011-11-18

**Authors:** Blaine A Christiansen, David L Kopperdahl, Douglas P Kiel, Tony M Keaveny, Mary L Bouxsein

**Affiliations:** 1 Center for Advanced Orthopedic Studies, Beth Israel Deaconess Medical CenterBoston, MA, USA; 2 Department of Orthopaedics, University of CaliforniaDavis, CA, USA; 3 O.N. DiagnosticsBerkeley, CA, USA; 4 Institute for Aging Research, Hebrew Senior Life, Harvard Medical School, Department of Medicine Beth Israel Deaconess Medical CenterBoston, MA, USA; 5 Departments of Mechanical Engineering and Bioengineering, University of CaliforniaBerkeley, CA, USA

**Keywords:** VERTEBRAL FRACTURE, FINITE ELEMENT ANALYSIS, QUANTITATIVE COMPUTED TOMOGRAPHY, BONE LOSS, VERTEBRAL STRENGTH, BONE STRENGTH, BIOMECHANICS

## Abstract

The biomechanical mechanisms underlying sex-specific differences in age-related vertebral fracture rates are ill defined. To gain insight into this issue, we used finite element analysis of clinical computed tomography (CT) scans of the vertebral bodies of L3 and T10 of young and old men and women to assess age- and sex-related differences in the strength of the whole vertebra, the trabecular compartment, and the peripheral compartment (the outer 2 mm of vertebral bone, including the thin cortical shell). We sought to determine whether structural and geometric changes with age differ in men and women, making women more susceptible to vertebral fractures. As expected, we found that vertebral strength decreased with age 2-fold more in women than in men. The strength of the trabecular compartment declined significantly with age for both sexes, whereas the strength of the peripheral compartment decreased with age in women but was largely maintained in men. The proportion of mechanical strength attributable to the peripheral compartment increased with age in both sexes and at both vertebral levels. Taken together, these results indicate that men and women lose vertebral bone differently with age, particularly in the peripheral (cortical) compartment. This differential bone loss explains, in part, a greater decline in bone strength in women and may contribute to the higher incidence of vertebral fractures among women than men. © 2011 American Society for Bone and Mineral Research.

## Introduction

Women have a higher incidence of osteoporotic fractures than men, over 25% of which are vertebral fractures.(1) Despite the high rate of occurrence and the significant personal and societal costs, the biomechanical mechanisms underlying vertebral fractures remain largely unknown.(2,3) It is possible that in addition to a decline in bone density, there are structural and/or geometric changes to the cortical and trabecular compartments with age that differentially affect men and women, making women more susceptible to vertebral fractures.

With age, vertebral trabecular bone begins to deteriorate, starting in the center of the vertebral body and progressing superiorly and inferiorly, with thinning of the endplates and cortical shell due to endosteal bone resorption.(4) Meanwhile, the cross-sectional area of the vertebral body increases with age in both men and women because of periosteal bone formation.(5,6) It is likely that these age-related changes in bone structure alter the mechanical contributions of the cortical and trabecular compartments of vertebral bodies, with the cortical compartment assuming a proportionally higher contribution in older subjects than in young subjects.(7,8) To date, several studies have used quantitative computed tomography (QCT)–based finite element analysis (FEA) to determine the contributions of cortical and trabecular bone to the strength of the distal radius,(9) proximal femur,(10–12) and vertebral body.(13,14) However, no studies have investigated the mechanical contributions of the bone compartments in subjects taken from a community-based study or have investigated how age and sex influence the mechanical role of trabecular and cortical bone in the thoracic and lumbar spine. Improved understanding of cortical and trabecular bone contributions to vertebral strength may guide efforts at diagnosing vertebral fragility and may enhance our understanding of therapies with differential effects on cortical vs. trabecular bone.

Conventional assessment of BMD in the spine typically analyzes only vertebrae of the lumbar region (typically L2–L4 or L1–L4), yet many fractures occur in the thoracic spine. How vertebrae from different regions of the spine lose bone with age is not well defined. Heterogeneity of age-related bone loss along the spine may contribute to higher incidence of vertebral fracture at some vertebral levels; therefore, it is possible that clinical fracture risk assessment can be improved by assessing vertebral levels in both the thoracic and lumbar spine.

In this study we used QCT-based FEA of lumbar (L3) and thoracic (T10) vertebrae of young men and women and old men and women to estimate vertebral body strength and its determinants (ie, bone density and morphology). We quantified age-related differences in the mechanical strength, bone strength, and bone density of cortical and trabecular bone compartments and determined whether these age-related differences are similar in vertebrae from the thoracic and lumbar spine and for men and women.

## Methods

### Subjects and Scan Parameters

Subjects were chosen from participants in the community-based Framingham Heart Study Offspring and Third Generation Multidetector CT Study.(15–18) The sample consisted of 30 men aged 35 to 42 years, 30 women aged 36 to 41 years, 30 men aged 73 to 82 years, and 30 women aged 74 to 83 years (Table 1). The study protocol was approved by the Boston University School of Medicine and Hebrew Senior Life, and all subjects gave written informed consent. The study is overseen by an independent data safety and monitoring board. For each subject, finite element models were created for the vertebral bodies of the T10 and L3 vertebrae, excluding the transverse and posterior elements. If the T10 or L3 vertebral body was fractured or missing from the QCT scan volume, an adjacent vertebral body was analyzed instead (Table 1).

**Table 1:** Subject Characteristics (mean ± standard deviation)

	*n*	Age (yrs)	Height (cm)	Mass (kg)
Young men	30	38.0 ± 1.8	179.4 ± 7.0	84.8 ± 12.2
Old men	30	78.0 ± 2.4	173.0 ± 6.5	83.5 ± 13.6
Young women	30	39.6 ± 0.9	164.9 ± 6.5	64.4 ± 10.4
Old women	30	77.6 ± 2.2	156.9 ± 6.5	62.6 ± 11.8

L2 was analyzed in one man (age 75) and one woman (age 77), T9 was analyzed in six women (ages 39, 40, 40, 41, 77, and 81), and T11 was analyzed in one woman (age 77). One woman (age 41) had no lumbar scan available, so only T10 was analyzed.

Scans were acquired during a 33-month period using the same eight-detector helical QCT scanner (Lightspeed Plus, General Electric, Milwaukee, WI, USA) at 120 kVp, 100–360 mAs. A chest scan imaged the area from the tracheal bifurcation to the base of the heart (approximately vertebral levels T7–T11), while an abdominal scan imaged a 150-mm-long volume superior to the upper endplate of S1 (approximately vertebral levels L2–L5). Scans had a nominal in-plane voxel size of 0.68 mm and a slice thickness of 2.5 mm. A multichambered hydroxyapatite phantom (Image Analysis, Columbia, KY, USA) was included in each scan to allow conversion of Hounsfield units to bone density (mg-HA/cm^3^).

### Finite Element Models

**Fig. 1:**
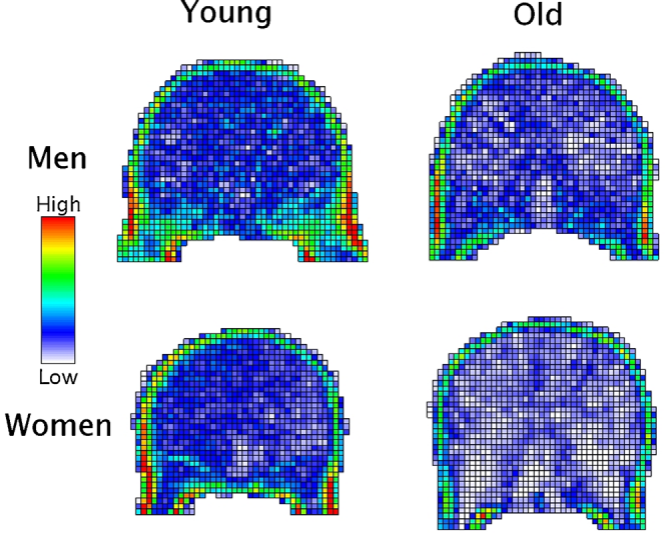
QCT-based finite element models of L3 vertebral bodies from a 38-year-old man (*top left*), 75-year-old man (*top right*), 40-year-old woman (*bottom left*), and 79-year-old woman (*bottom right*). Each vertebra (excluding posterior elements) was segmented from the QCT image, rotated into a standard coordinate system, and resampled into 1-mm cube-shaped voxels. The finite element mesh was created by converting each voxel into an 8-noded brick element. Elastic-perfectly plastic material properties were assigned to each element using the mineral density derived from the brightness of the voxel along with the empirical correlations between mechanical properties and calibrated BMD for human vertebral trabecular bone.(22) Images are representative of the means for peripheral bone mass.

**Fig. 2:**
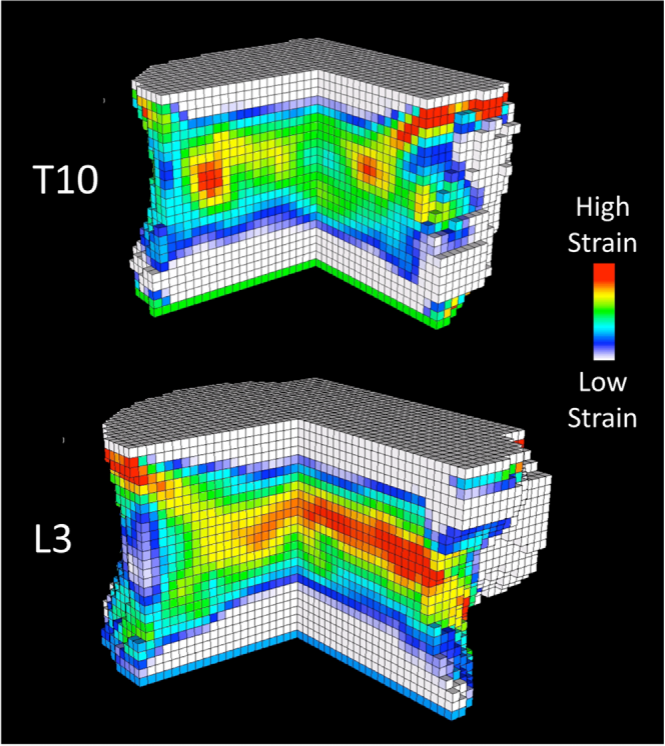
Finite element models of vertebral bodies loaded in axial compression to 2% strain (applied displacement over total height). A thin layer of polymethyl methacrylate (PMMA) was virtually applied to the endplates to simulate conditions of experimental testing. Material failure of the bone was modeled by a von Mises failure criterion. Because failure strain is relatively independent of bone density, contour plots of strain indicate predicted regions of failure.

QCT-based finite element models of T10 and L3 vertebrae were generated for each patient using previously published methods.(19–21) Briefly, each vertebra (excluding posterior elements) was segmented from the image, rotated into a standard coordinate system, and resampled into 1-mm cube-shaped voxels. The finite element mesh was created by converting each voxel into an 8-noded brick element (Fig. 1). Elastically anisotropic(21) and elastic-perfectly plastic material properties were assigned to each element using the QCT mineral density of the voxel along with the empirical correlations between mechanical properties and calibrated BMD for human vertebral trabecular bone.(22) Material failure of the bone was modeled by a von Mises failure criterion. A thin layer of polymethyl methacrylate (PMMA) was virtually applied to the endplates to simulate conditions of experimental testing.(23) We applied uniform compressive displacement boundary conditions to the external surfaces of these PMMA layers and computed the axial stiffness (N/mm) and compressive strength (N) of the vertebra, taken to be the total reaction force generated at an imposed displacement equivalent to an overall bone compressive strain of 2% (applied displacement divided by bone height) (Fig. 2). Cadaver studies using this approach have shown strong correlations with experimentally measured vertebral strength.(21,24)

To gain insight into the biomechanical mechanisms underlying age-related differences in vertebral strength, we conducted parametric studies where key parameters from each finite element model were varied one at a time and the strength estimates recomputed to determine the effects of these parameters on vertebral strength (Table 2). First, to delineate the influence of geometry on vertebral strength, an arbitrary constant density (100 mg/cm^3^) was applied to all voxels across all FE models, and the resulting vertebral strength was considered “geometric strength”—a measure of the effect of vertebral geometry independent of differences in tissue density. Although the magnitude of geometric strength is dependent on the arbitrary constant density chosen (100 mg/cm^3^), differences between groups will be maintained regardless of this value. Second, the peripheral 2 mm of bone was removed, the vertebrae virtually compressed again, and the resulting strength estimate termed the “trabecular strength.” The difference between the total vertebral strength and trabecular strength was defined as the “peripheral Strength.” The term peripheral strength is used rather than “cortical strength” because the outer 2 mm of bone contains both the real cortical shell (about 0.4 mm thick) and the adjacent trabeculae that would be unloaded upon removal of the cortical shell.(13) We also computed the ratio of trabecular strength to total vertebral body strength. In addition to these compressive strength measures, we computed the mechanical response to anterior bending by applying a pure bending rotation (1^o^) to the superior endplate and computed the bending stiffness and the ratio of axial to bending stiffness.(25) To gain an understanding of overall failure stress, we measured the average cross-sectional area (CSA) for the entire vertebral body and computed an average failure stress as the ratio of failure strength to CSA.(14)

**Table 2:** Definitions of Outcome Variables for Finite Element Analysis

Variables	Definition
Strength variables	
Vertebral body strength	Strength of the vertebral body under compressive loading conditions.
Geometric strength	Compressive strength after removal of all intra- and intervertebral bone density effects. All vertebrae are assigned the same “reference” bone density (100 mg/cm^3^). This is a measure of how vertebral geometry alone influences compressive strength.
Trabecular strength	Compressive strength of the trabecular compartment. The peripheral 2 mm layer of bone (primarily consisting of the cortical shell) is removed and the strength of the remaining trabecular bone is found.
Peripheral strength	Quantifies the contribution of vertebral strength primarily due to the cortical compartment (ie, the peripheral 2 mm layer of bone). Calculated as vertebral body strength–trabecular strength.
Bending stiffness	Vertebral bending stiffness when the bone is subjected to an anterior-posterior (AP) bending moment.
Axial stiffness	Vertebral compressive stiffness when the bone is subjected to a compressive force.
Density and mass variables	
Vertebral body density	Average bone mineral density of the entire vertebral body including both cortical and trabecular bone.
Vertebral body mass	Total bone mineral mass of the entire vertebral body.
Trabecular density	Average bone mineral density of the trabecular compartment.
Trabecular mass	Total bone mineral mass of the trabecular compartment.
Peripheral density	Average bone mineral density of the peripheral 2 mm of bone (cortical compartment)
Peripheral mass	Total bone mineral mass of the peripheral 2 mm of bone (cortical compartment)
Average CSA	Mean cross-sectional area of the vertebral body
Ratios	
Vertebral body strength/vertebral body density	Quantifies the strength per unit of volumetric bone mineral density. A relatively high value indicates that the vertebra is relatively strong after accounting for average bone density effects.
Trabecular strength/vertebral body strength	Quantifies the relative biomechanical role of the trabecular compartment. A ratio of 0.40, for example, implies that 40% of the overall vertebral strength is attributable to the trabecular compartment.
Bending stiffness/Axial stiffness	This quantifies the resistance to AP bending loads relative to compressive loads. A low ratio signifies a bone having a relatively low resistance to bending compared to its resistance to compression, indicating a propensity to fail under AP bending type loads.
Vertebral body strength/Average CSA	The failure stress averaged over the entire vertebral body for axial compression loading.

### Statistical analysis

Two-factor ANOVA with repeated measures for T10 and L3 was used to determine age-, sex-, and vertebral level–related differences in vertebral body strength and other related outcomes. In addition, where significant interactions between factors were identified, we used unpaired *t*-tests to compare men and women, young and old groups, and thoracic and lumbar regions. Differences were considered significant for *p* < .0125 due to Bonferroni correction for three independent hypotheses. Correlation between thoracic and lumbar strength values was performed using paired data for all subjects. *R*^*2*^ values were calculated for all subjects and for each sex and age group.

## Results

### Differential age-related declines in vertebral body mass and density for men and women

**Fig. 3:**
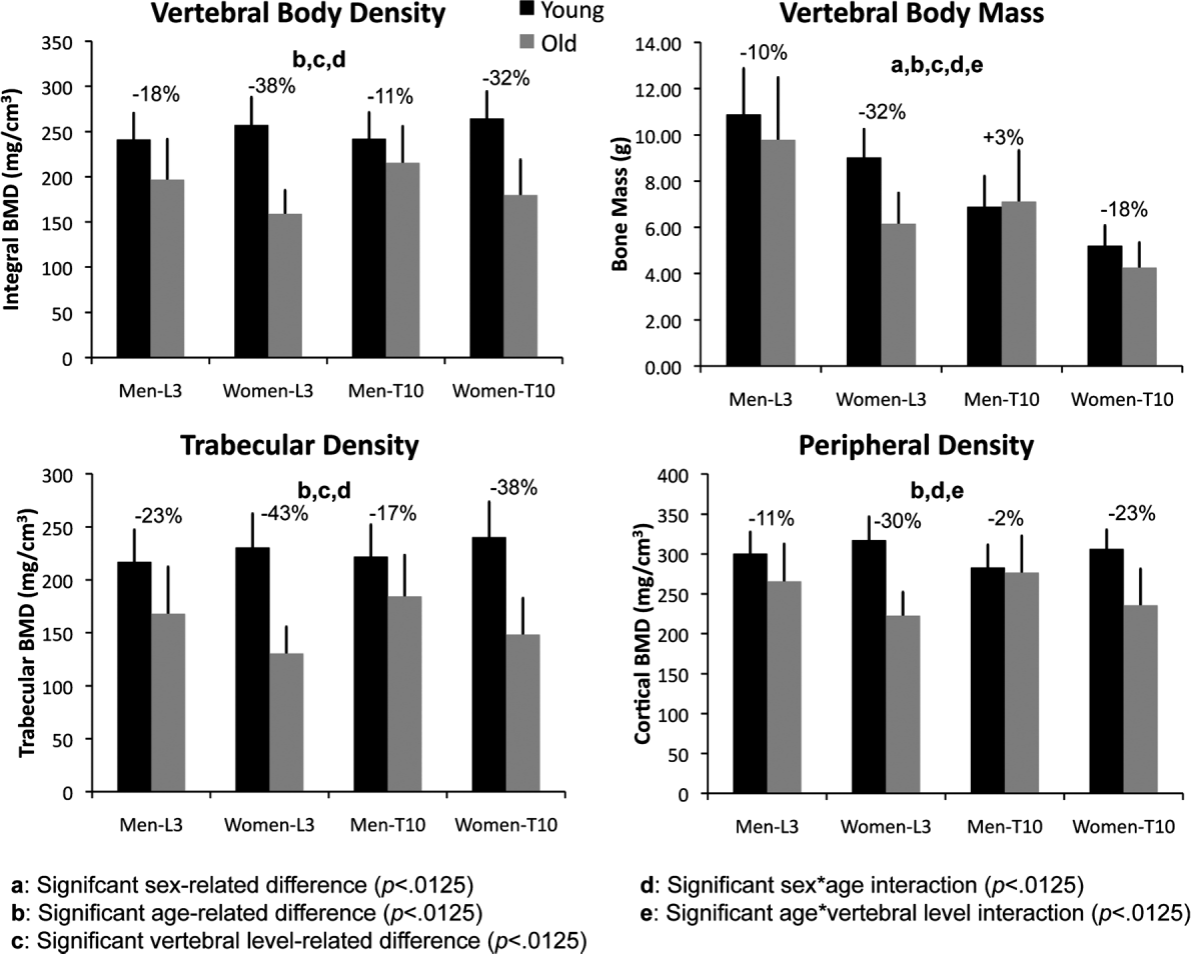
Results for density and mass variables. Bone mass and density declined with age in both the peripheral (cortical) and trabecular compartments of T10 and L3, with women exhibiting significantly greater losses than men. Total vertebral body density declined significantly more with age in women than in men, with declines in both trabecular and peripheral bone density for women, while men had smaller declines in trabecular bone density and either no decline or only a small decline in peripheral density.

Bone mass and density declined with age in both the peripheral (cortical) and trabecular compartments of T10 and L3, with women exhibiting significantly greater losses than men (Table 3, Fig. 3). For example, total vertebral body density declined 2- to 3-fold more with age in women (−32% at T10, −38% at L3) than men (−11% at T10, −18% at L3). This decrease in total density was associated with declines in both trabecular (−38% at T10, −43% at L3) and peripheral (−23% at T10, −30% at L3) bone density for women, while men had smaller declines in trabecular bone density (−17% at T10, −23% at L3) and either no decline (T10) or only a small decline (−11% at L3) in peripheral density. Thus, total vertebral body mass was largely maintained with advancing age in men, because of small decreases in trabecular mass (−4% at T10, ns; −15% at L3, *p* = .01), and either no change (L3) or increases in peripheral mass (+14% at T10, *p*= .03).

**Table 3:** Results of Finite Element Analysis (mean ± standard deviation)

	L3						T10						
			
	Young men (*n=* 30)	Old men (*n=* 30)	% Difference: old vs. young	Young women (*n=* 30)	Old women (*n=* 30)	% Difference: old vs. young	Young men (*n=* 30)	Old men (*n=* 30)	% Difference: old vs. young	Young women (*n=* 30)	Old women (*n=* 30)	% Difference: old vs. young	Statistical significance
Strength variables													
Vertebral body strength (N)	9647 ± 2205	7010 ± 2735	−27.3%^†^	8503 ± 1413	4046 ± 1091^‡^	−52.4%^†^	7759 ± 1800	6288 ± 2200	−19.0%^†^	6473 ± 1291^‡^	3617 ± 1135^‡^	−44.1%^†^	abcde
Geometric strength (N)	3504 ± 547	3902 ± 589	+11.4%^†^	2778 ± 301^‡^	3079 ± 450^‡^	+10.8%^†^	2735 ± 394	3025 ± 477	+10.6%	2017 ± 304^‡^	2318 ± 332^‡^	+14.9%^†^	abc
Trabecular strength (N)	5651 ± 1537	3375 ± 1697	−40.3%^†^	4853 ± 986	1757 ± 670^‡^	−63.8%^†^	4763 ± 1272	3369 ± 1461	−29.3%^†^	3724 ± 908^‡^	1788 ± 658^‡^	−52.0%^†^	abce
Peripheral strength (N)	3996 ± 726	3635 ± 1237	−9.1%	3649 ± 502	2289 ± 508^‡^	−37.3%^†^	2997 ± 603	2919 ± 869	−2.6%	2748 ± 416	1829 ± 529^‡^	−33.4%^†^	abcd
Bending stiffness (kNm/rad)	3.61 ± 1.19	3.40 ± 1.44	−5.9%	2.63 ± 0.47^‡^	1.74 ± 0.54^‡^	−33.9%^†^	3.27 ± 1.06	3.37 ± 1.58	+3.0%	2.16 ± 0.62^‡^	1.65 ± 0.53^‡^	−23.4%^†^	ab
Axial stiffness (kN/mm)	37.8 ± 7.7	29.4 ± 9.6	−22.0%^†^	33.7 ± 4.3	18.8 ± 4.4^‡^	−44.2%^†^	36.5 ± 7.4	30.2 ± 7.6	−17.1%^†^	32.0 ± 5.7^‡^	20.4 ± 5.4^‡^	−36.3%^†^	abd
Density and mass variables													
Vertebral body density (mg/cm^3^)	241 ± 29	197 ± 45	−18.4%^†^	257 ± 30	159 ± 26^‡^	−38.2%^†^	242 ± 29	216 ± 40	−11.0%^†^	265 ± 30^‡^	180 ± 39^‡^	−32.0%^†^	bcd
Vertebral body mass (g)	10.89 ± 1.98	9.80 ± 2.68	−10.1%	9.03 ± 1.23^‡^	6.16 ± 1.33^‡^	−31.7%^†^	6.89 ± 1.33	7.12 ± 2.21	+3.3%	5.20 ± 0.88^‡^	4.26 ± 1.08^‡^	−18.2%^†^	abcde
Trabecular density (mg/cm^3^)	217 ± 30	168 ± 44	−22.6%^†^	231 ± 32	130 ± 25^‡^	−43.4%^†^	222 ± 30	184 ± 39	−16.9%^†^	240 ± 33	148 ± 34^‡^	−38.3%^†^	bcd
Trabecular mass (g)	6.94 ± 1.42	5.87 ± 1.76	−15.4%^†^	5.61 ± 0.88^‡^	3.50 ± 0.90^‡^	−37.5%^†^	4.21 ± 0.87	4.05 ± 1.39	−3.8%	3.00 ± 0.61^‡^	2.25 ± 0.60^‡^	−25.0%^†^	abcde
Peripheral density (mg/cm^3^)	301 ± 27	266 ± 47	−11.5%^†^	317 ± 29	223 ± 30^‡^	−29.8%^†^	283 ± 29	277 ± 46	−2.2%	306 ± 24^‡^	236 ± 46^‡^	−23.1%^†^	bde
Peripheral mass (g)	3.95 ± 0.57	3.92 ± 0.96	−0.7%	3.42 ± 0.37^‡^	2.66 ± 0.46^‡^	−22.3%^†^	2.68 ± 0.48	3.07 ± 0.85	+14.4%	2.21 ± 0.28^‡^	2.01 ± 0.50^‡^	−9.0%	acde
Average CSA (cm^2^)	11.81 ± 1.29	12.60 ± 1.48	+6.7%	10.57 ± 1.02^‡^	10.58 ± 1.65^‡^	+0.1%	8.99 ± 1.35	9.67 ± 1.75	+7.6%	6.86 ± 1.03^‡^	7.60 ± 1.29^‡^	+10.8%	ac
Ratios													
Vertebral body strength/vertebral body density (Ncm^3^/mg)	39.8 ± 6.3	34.6 ± 6.5	−13.0%^†^	33.0 ± 3.4^‡^	25.1 ± 3.8^‡^	−23.8%^†^	31.8 ± 4.5	28.7 ± 5.4	−9.7%	24.4 ± 3.5^‡^	19.8 ± 3.1^‡^	−18.8%^†^	abc
Trabecular strength/vertebral body strength	0.58 ± 0.03	0.47 ± 0.07	−19.3%^†^	0.57 ± 0.03	0.42 ± 0.06	−25.2%^†^	0.61 ± 0.03	0.53 ± 0.06	−13.3%^†^	0.57 ± 0.04^‡^	0.49 ± 0.05^‡^	−14.6%^†^	abce
Bending stiffness/axial stiffness (mm^2^/rad)	94.0 ± 15.8	114.3 ± 23.5	+21.6%^†^	78.2 ± 9.4^‡^	92.5 ± 16.2^‡^	+18.3%^†^	88.6 ± 17.2	108.8 ± 23.8	+22.8%^†^	66.7 ± 11.3^‡^	80.4 ± 11.0^‡^	+20.6^†^%	abc
Vertebral body strength/Ave. CSA (MPa)	8.16 ± 1.43	5.51 ± 1.86	−32.5%^†^	8.14 ± 1.48	3.88 ± 1.06^‡^	−52.3%^†^	8.65 ± 1.62	6.59 ± 1.71	−23.8%^†^	9.61 ± 1.67	4.70 ± 1.49^‡^	−51.1%^†^	bcd

Main effect: a: sex-related difference; b: age-related difference; c: vertebral level-related difference (*p* < .0125).

Interactions: d: sex–age interaction; e: age–vertebral level interaction (*p* < .0125).

Post-hoc analysis: ^†^significant difference between old and young (same sex and vertebral level, *p* < .0125); ^‡^significant difference between women and men (same age and vertebral level, *p* < .0125).

### Differential age-related declines in vertebral strength for men and women

**Fig. 4:**
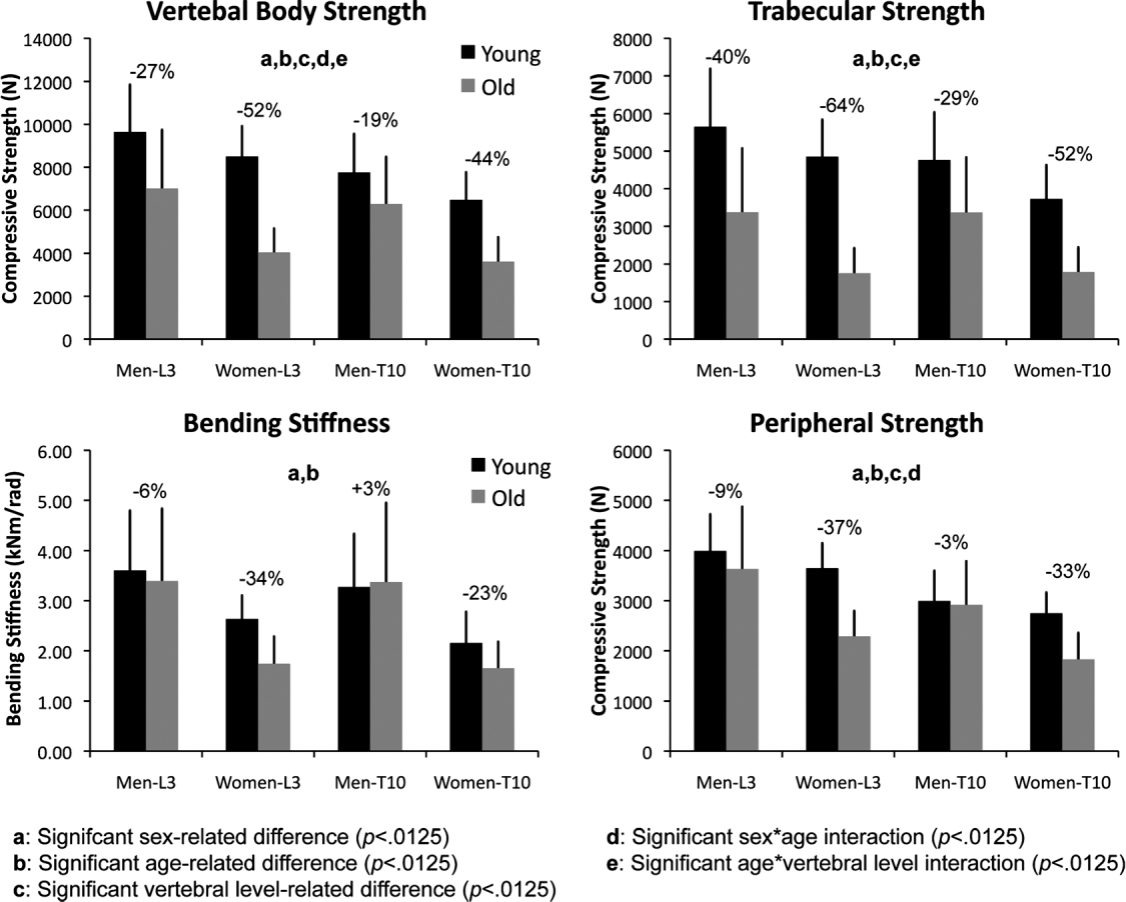
Results for strength variables. Vertebral body strength declined with age for both men and women, with women exhibiting significantly greater losses of strength than men. Trabecular strength declined significantly for both sexes, while peripheral strength declined 4- to 10-fold more with age in women than in men. Similarly, bending stiffness declined significantly with age in women but did not change in men.

**Fig. 5:**
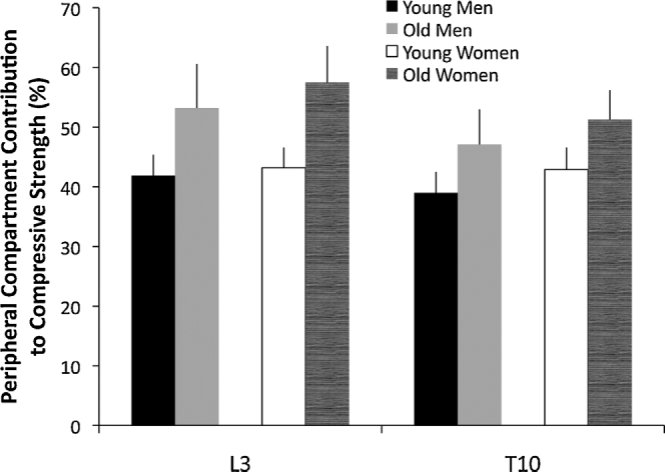
The proportion of vertebral strength attributable to the peripheral compartment increased with age from 43% to 57% for L3 and 43% to 51% for T10 in women and from 42% to 53% for L3 and 39% to 47% for T10 in men (no significant difference between men and women).

Vertebral strength outcomes declined with age for both men and women, with women exhibiting significantly greater losses of strength than men (Table 3, Fig. 4). For example, vertebral compressive strength decreased 2-fold more with age in women (−44% at T10, −52% at L3) than in men (−19% at T10, −27% at L3; *p* = .0008). Trabecular strength declined significantly and similarly for both sexes (−52% at T10, −64% at L3 for women; −29% at T10, −40% at L3 for men), whereas peripheral strength declined 4- to 10-fold more with age in women (−33% at T10, −37% at L3) than in men (-3% at T10, -9% at L3; *p* < .0001). As a result, the proportion of vertebral strength attributable to the peripheral compartment increased significantly with age, from 43% to 57% for L3 and 43% to 51% for T10 in women and from 42% to 53% for L3 and 39% to 47% for T10 in men (Fig. 5; no significant difference between men and women). geometric strength increased 11 to 15% with age at both T10 and L3 for both women and men because of increased vertebral body size.

Axial stiffness declined with age in both sexes but decreased more in women (−36% at T10, −44% at L3) than in men (−17% at T10, −22% at L3; *p* = .0007). In contrast, bending stiffness declined significantly with age in women (−23% at T10, −34% at L3) but did not change in men (+3% at T10, −6% at L3).

### Correlation between strength measurements of lumbar and thoracic vertebrae

**Fig. 6:**
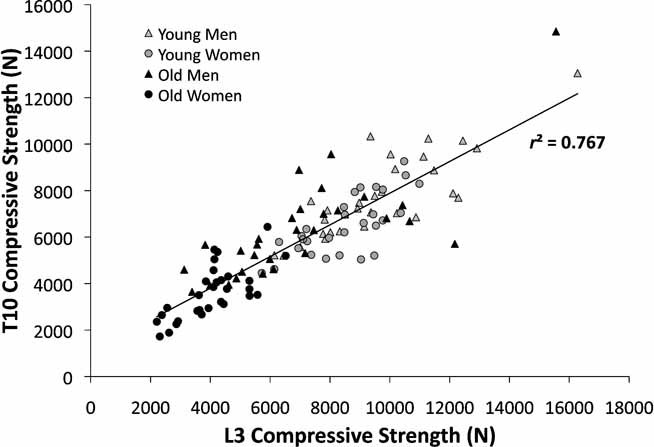
There was a moderately strong correlation between compressive strength values for L3 and T10 when all subjects were considered together (*r^2^* = 0.77). When each age-sex group was plotted independently, the correlation between vertebral body strength for L3 and T10 was higher for men than for women and higher for young subjects than for old subjects (*r^2^* = 0.69 for young men, 0.59 for old men, 0.55 for young women, 0.50 for old women).

There was a moderately strong correlation between compressive strength values for L3 and T10 when all subjects were considered together (*r*^*2*^ = 0.77, Fig. 6). When each age-sex group was plotted independently, the correlations between vertebral body strength for L3 and T10 were lower than when all subjects were considered together, and they were higher for men than for women and higher for young subjects than old subjects, such that in older women, only 50% of the variability in T10 strength was explained by L3 strength (*r*^*2*^ = 0.69 for young men, *r*^*2*^ = 0.59 for old men, *r*^*2*^ = 0.55 for young women, *r*^*2*^ = 0.50 for old women).

## Discussion

In this study we used QCT-based FEA of lumbar and thoracic vertebrae of young and old men and women to determine age-related changes in mechanical strength, bone mass, and bone density of cortical and trabecular bone compartments. As expected, vertebral strength decreased with age for both men and women, but it decreased more dramatically in women than in men because of a greater decline in bone mass in both trabecular and peripheral bone compartments. Notably, in men there was little age-related decline in peripheral bone strength. These results provide evidence of a different compartment-specific pattern of age-related decline in vertebral bone mass and strength in women vs. men that may contribute to the higher incidence of vertebral fractures among women.

As expected, compressive strength predicted by finite element analysis was higher in men than women, and higher in L3 than T10, both of which can largely be explained by differences in bone size. It has previously been shown that vertebral compressive failure loads are lower in women, but estimated failure stresses are similar in both sexes,(26,27) suggesting that vertebral size explains much of the difference in compressive failure loads between men and women. Our data support this, as both compressive strength and average vertebral cross-sectional area are larger in men than women, but no sex-related difference in estimated failure stress (vertebral body strength/average CSA) was observed. Similarly, previous studies(26,28–34) have reported variation in compressive strength of human cadaveric vertebrae along the thoracic and lumbar spine, with an increase in vertebral compressive failure load and a decrease in estimated failure stress (failure load / average vertebral cross-sectional area) from the thoracic to lumbar spine.(26,29,30,32) We observed a similar pattern, because T10 failure stress was higher than L3 failure stress for all groups. For these calculations we used the average CSA of the vertebral bodies. It is possible that minimum CSA instead of average CSA would yield different results for estimates of failure stress. Unfortunately, we are unable to calculate minimum CSA using our current software. However, we predict that differences observed between young and old and between thoracic and lumbar vertebrae will be maintained whether we normalize by average CSA or minimum CSA. In the absence of minimum CSA measures, geometric strength can provide similar information, because it is a strength measure that is wholly dependent on geometry (and presumably minimum CSA).

In contrast to sex-specific differences, age-related differences in compressive strength cannot be explained by changes in bone size but rather are due primarily to changes in bone mass and density. Geometric strength, a measure of the isolated contribution of bone geometry to compressive strength, was higher in old subjects than in young subjects, indicating that *considering only bone size/geometry*, older subjects have stronger vertebrae than young subjects, but this age-related increase in geometric strength was generally small and did not offset age-related declines in overall vertebral strength. This finding is supported by previous studies that have shown an increase in cross-sectional area of vertebral bodies with age.(5,6) However, it is well established that volumetric bone density (vBMD) declines in both men and women with age, resulting in an overall loss of vertebral body strength. Previous studies have shown that vBMD is similar in young men and women,(35,36) and may even be slightly higher in women,(36) but that women clearly exhibit a greater age-related decline in vBMD and compressive strength at the lumbar spine than men.(36) Our study confirms these prior observations, because young men and women had similar vBMD values, yet the women exhibited significantly greater age-related declines in bone mass, density, and strength than men.

Age-related decline in vertebral compressive strength in men can be attributed almost exclusively to a decline in trabecular strength, because peripheral strength and density were largely maintained. In women, changes in both the trabecular and the peripheral compartment contribute to the age-related loss of strength, with a relatively larger loss in the trabecular compartment. As a result, the percentage of total bone strength attributable to the peripheral compartment increases with age in both men and women. Altogether, these data suggest that sex-specific differences in the age-related changes in cortical bone contribute to the lower incidence of vertebral fractures in men than in women. The finding that bending stiffness significantly decreased with age in women (−23% at T10, −34% at L3) but not in men (+3% at T10, −6% at L3) is likely due to differences in bone loss from the peripheral compartment. These differences in the peripheral compartment may result in a decreased resistance to loads induced by forward flexion for women relative to men, making women more vulnerable to sustaining wedge fractures.

The role of vertebral osteophytes must also be considered when interpreting the bone mass and strength changes in the peripheral compartment. Osteophytes are not specifically removed by the image processing used in this study and are therefore included in the peripheral bone measurements. Inclusion of osteophytes in the peripheral compartment may mask underlying age-related declines in bone mass and strength. In addition, because the peripheral compartment is defined as the outer 2 mm of bone in this study, areas with large osteophytes may cause the trabecular compartment to include some regions of cortical bone. One study reported a slightly higher prevalence of vertebral osteophytosis in men than in women older than age 50 (84% vs. 74%, respectively), although the distribution of osteophytes along the spine was similar in both sexes.(37) In contrast, another study reported a higher prevalence of vertebral osteophytosis in women than in men.(38) Altogether, these epidemiologic studies do not indicate a marked difference in the prevalence of osteophytes by sex, thus limiting the confounding role of osteophytes on sex-specific differences observed in the current study. Nonetheless, further studies may be needed to delineate compartment-specific changes in bone mass and bone strength without the possible confounding contribution of osteophytes.

Conventional assessment of spine BMD typically analyzes only vertebrae of the lumbar region (typically L2–L4 or L1–L4), yet many fractures occur in the thoracic spine. To estimate the error in predicting thoracic vertebral strength measurements from lumbar analyses, we determined the association between FE-determined lumbar and thoracic vertebral strength. We found a strong correlation between compressive strength estimates for T10 and L3 (*r*^*2*^ = 0.77 for all subjects), but when each age and sex group was considered individually, we found that the association was weaker in old vs. young subjects and also weaker in women vs. men (ie, *r*^*2*^ = 0.50 for old women). Similarly, Bürklein et al.(31) compared the compressive strength of T6, T10, and L3 vertebrae in 119 cadavers and reported only modest correlations between the different levels (eg, *r*^*2*^ = 0.46 for T10 vs. L3). These results indicate that there is heterogeneity of vertebral strength along the spine. It remains to be determined whether clinical fracture risk assessment can be improved by assessing vertebral levels in both the thoracic and lumbar spine.

This study had several strengths that are novel contributions to study of vertebral fractures. First, we analyzed an age-stratified set of subjects from a community-based population. Therefore, the observed trends should reflect typical changes that occur in the population in general, although the racial representation for this study was primarily white people. Second, the use of finite element analysis and the controlled parameter studies enabled us to simulate different loading conditions and isolate the contributions of the trabecular and peripheral compartments to the strength of the whole bone. This provided unique insight into the role of the trabecular and peripheral compartments, which would be difficult to achieve using simpler structural models based on beam-and-column theories.

This study also had several limitations. First, the study was cross-sectional, and therefore age-related “changes” reported for bone strength or other contributing factors were inferred based on cross-sectional differences between young and old subjects. Second, the peripheral density and strength measurements included the outside 2 mm of bone, which contained trabecular as well as cortical bone, including osteophytes. In young subjects, the cortical shell of the vertebral bodies is approximately 400–500 µm thick and decreases to only 200–300 µm in elderly individuals.(39,40) Ideally, to observe differences between cortical and trabecular bone, the peripheral shell would contain only cortical bone, but the spatial resolution of these clinical scans precludes accurate segmentation of this thin cortex. However, our previous micro-CT-based finite element analysis of T10 vertebrae, which captured the cortical shell at high resolution, have shown that the cortical shell supports approximately 40–50% of the compressive load.(41) This is consistent with the load-sharing estimates of the peripheral bone in the current study, providing a degree of validation to these model predictions. A third limitation is that our sample size was modest (*n* = 30 subjects/group), although it was adequately large for us to find significant differences for all variables examined. Fourth, the finite element models were loaded via PMMA plates at the top and bottom of the vertebral body, as is commonly done in cadaver studies. Again, our prior studies using micro-CT-based FEA have shown that overall load-sharing trends are relatively insensitive to the presence of a disc(41) and thus we would not expect our reported trends to differ notably with an intervertebral disc instead of PMMA at the endplates. Finally, the method used to assess “peripheral” properties (eg, total strength – trabecular strength) ignores load sharing between the two compartments. A thorough analysis of load sharing between trabecular and cortical bone would require a high-resolution micro-CT-based analysis.(41) Unfortunately, because of the resolution used to obtain the CT scans in this study, this type of analysis was not possible. However, the contributions of the individual compartments that we calculate with continuum models in the current study is consistent with what Eswaran reported with the micro-CT-based models, which suggests that by taking off the 2 mm of bone, we are effectively removing the cortical shell (about 0.4 mm thick) and adjacent trabeculae that would be unloaded upon removal of the cortical shell. Therefore, we conclude that removal of the outer 2 mm in the continuum models provides a good estimate of the results that would be obtained with removal of just the real cortical shell—because for the latter, the adjacent trabeculae become unloaded since there is no cortical shell to transmit load in the vertical direction to and from these trabeculae.(13)

## Conclusions

Decreases in vertebral strength occur differently with age for men and women, particularly in the peripheral (cortical) compartment. Whereas women lost bone mass and bone strength in both the cortical and trabecular compartments with age, men primarily lost bone mass and strength from the trabecular compartment, while cortical bone properties did not decrease with age. Combined with the increased mechanical role of the cortical compartment with age, this presents a potential mechanism that may contribute to the disparate incidence of vertebral fractures in women and men.
